# Mother’s Croup Reveals That Parainfluenza Virus Is the Cause of Her Son’s Viral Parotitis

**DOI:** 10.7759/cureus.54201

**Published:** 2024-02-14

**Authors:** John W Green, Spencer W Green

**Affiliations:** 1 Hospital Medicine, Rural Physicians Group, Magruder Hospital, Port Clinton, USA; 2 Engineering, The Ohio State University, Columbus, USA

**Keywords:** mumps, causality assessment, croup, parainfluenza, viral parotitis

## Abstract

In modern practice viral parotitis is unlikely to be due to mumps. Case and surveillance studies have detected a host of other viruses in mumps-negative viral parotitis, but because of their weak association with viral parotitis, it has been difficult to establish causality. This case report is unique because a familial pair presented in tandem with different manifestations of an infection with the parainfluenza virus. These circumstances allowed the strong association of the parainfluenza virus with the mother’s croup to be substituted for the normally weak association of the parainfluenza virus with the son’s viral parotitis. This strongly inferred that the parainfluenza virus caused the patient’s viral parotitis and provides the best evidence to date of a virus other than mumps causing viral parotitis.

## Introduction

Prior to the licensure of the mumps vaccine in 1967 and its subsequent routine use in the United States, mumps was a universal childhood disease with more than 150,000 reported cases in 1968 [1,2]. Each case of viral parotitis was attributed to mumps because mumps dwarfed all other causes. Microbiologic testing could scarcely be recommended when it could not appreciably improve diagnostic accuracy or clinical outcomes. With the introduction of the mumps vaccine, cases of mumps dropped 600-fold and reached a nadir of 250 cases annually in the early 2000s [3]. This precipitous decline rendered mumps a rare disease, and a specific diagnosis for viral parotitis became necessary. Over the last 55 years, research into other viruses that cause viral parotitis has been limited to case or surveillance studies [3-10]. These types of reports cannot assign causality, they can only infer it through the strength of the association between the virus and the illness [11]. However, no virus, other than mumps, carries a strong association with parotitis, and, therefore, the ability of these studies to assign causality is limited. This case is unique in that a familial pair shared overlapping hospitalizations with bronchiolitis due to the parainfluenza virus, and both had an additional manifestation of parainfluenza infection that the other did not share. The son, who serves as the patient in this case report, had parotitis, and his mother had croup. The strong association of the parainfluenza virus with croup provided a strong inference of causality of the mother’s croup to the detected parainfluenza virus. Through logical reasoning, this strong inference of causality was transferred from the mother’s croup to the patient’s parotitis, which provides the strongest evidence to date that any virus other than mumps causes viral parotitis.

## Case presentation

**Figure 1:**
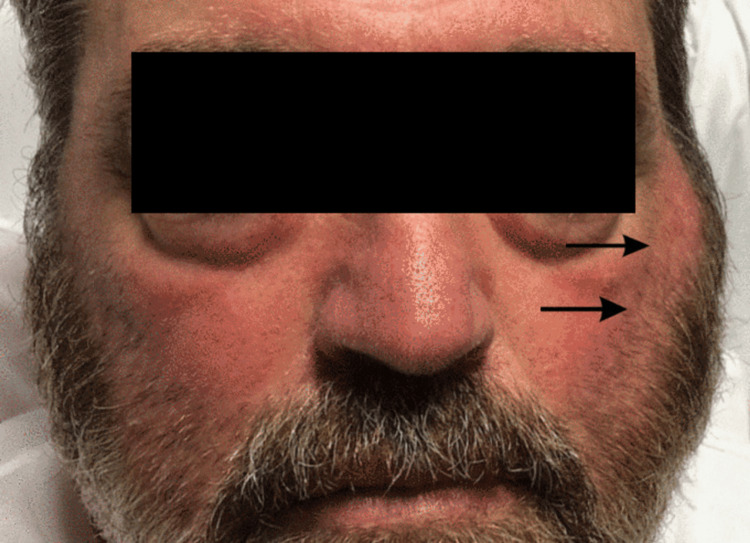
The patient with bilateral parotitis; however, only the edema of the left parotid gland can be observed.

A 64-year-old male with relapsed multiple myeloma presented to the emergency department with several days of severe cough and dyspnea on exertion. The patient had undergone an autologous stem cell transplant several years earlier. The patient’s current chemotherapy regimen included dexamethasone, pomalidomide, and carfilzomib, which had resulted in secondary hypogammaglobulinemia. The patient was started on bronchodilators and steroids and admitted to the hospital, where he expressed concern that he had exposed his mother to this respiratory infection. On exam, the patient had a fever, severe hacking cough, profound wheezing, and tachypnea. The patient was not hypoxemic. The oral exam did not show any purulent drainage from Stensen’s duct, mucositis, or dental caries. On day 3, the patient developed a painful lump on the left side of his neck that migrated toward his jaw. This fullness was suspected to be parotitis, so the patient was started on broad-spectrum antibiotics, blood cultures were obtained, and cross-sectional imaging was completed to look for an obstructing sialolith. Imaging did not show a sialolith, but it did support the clinical diagnosis of unilateral left-sided parotitis. On day 4, the patient developed painful swelling of the right side of his jaw (Figure 1). The patient’s now bilateral parotitis was far more likely to be viral in origin and concern immediately shifted to mumps. This concern was heightened by the determination that the patient had not been revaccinated against mumps since his autologous bone marrow transplant. Appropriate testing for mumps was ordered including serology, salivary culture, and rt-PCR testing on saliva and blood. The concern for mumps was shortly lived, however, the next day (3.7 days after the patient was admitted to the hospital) the patient’s mother was admitted to the same hospital for dyspnea on exertion, profound wheezing, hypoxemia, and a distinctly croupy cough. The mother’s illness cast significant doubt that their shared illness was due to mumps as she likely had natural immunity and would not have contracted mumps from her son; furthermore, the croupy cough and approximate four-day incubation period were indicative of a parainfluenza virus infection (parainfluenza virus has an incubation period of two to six days), which case and surveillance studies had shown was one of the most common viruses isolated in mumps-negative viral parotitis [3-10,12]. These findings made parainfluenza virus the suspected etiology of the shared illness. Respiratory PCR panels were promptly obtained on both patients, which returned dual positive results for parainfluenza virus type 3. Later, all testing for mumps resulted in a negative. For reasons that are unclear, the rt-PCR tests on blood and saliva were never completed.

## Discussion

The detection of parainfluenza virus type 3 and no other virus strongly supports the presumption that parainfluenza virus type 3 caused the patient's parotitis. The detection of parainfluenza virus type 3 by the Biofire Respiratory 2.1 Panel is unlikely to represent a false positive as the assay has demonstrated a specificity of 99.4% in the detection of parainfluenza virus type 3 [13]; furthermore, the presence of parainfluenza virus is unlikely to represent colonization as parainfluenza virus is generally a pathogen when isolated from the respiratory tract [14]. These findings, however, do not establish causation of the patient's parotitis by parainfluenza virus type 3.

The standard for causal inference in epidemiologic studies is the Bradford Hill Criteria [11]. These criteria were first suggested in 1965 to discern what aspects of an association between exposure and disease suggested causality [15]. Of the nine aspects included in the criteria, the most important one is the strength of the association between the exposure and the disease [15]. Since the association of parainfluenza virus and viral parotitis is weak, the detection of parainfluenza virus alone does not establish its causality of viral parotitis. In contrast to mumps, which carries a strong association with viral parotitis, where detection of the virus would have strongly inferred causality. This case report is unique because the familial pair share overlapping hospitalizations, where the patient’s parotitis is informed by the mother’s croupy cough. Since the parainfluenza virus has a strong association with croup, the causality of the mother’s croup can be strongly inferred from its detection. By establishing through inductive reasoning that both the patient and mother share the same infection and that this infection is responsible for the patient’s parotitis, this strong inference of causality can be transferred from the mother’s croup to the patient’s parotitis.

Inductive reasoning allows us to make a broader generalization based on several observations. The verifiable observations that the familial pair shared an epidemiologic link, had overlapping hospitalizations with severe bronchiolitis, and tested positive for the identical virus allows one to inductively reason that both individuals are infected with the same virus. This strongly infers that the patient is infected with the parainfluenza virus. Furthermore, the verifiable observations that the patient’s infection started just before the hospitalization and that the viral parotitis started just after the hospitalization allow one to inductively reason that the viral parotitis was caused by the patient’s infection. Since there is a strong inference that the mother’s illness is caused by the parainfluenza virus, that the patient is also infected with the same parainfluenza virus, and that this infection caused the viral parotitis, there is a strong inference of causality of the patient’s parotitis by the parainfluenza virus.

## Conclusions

In modern practice viral parotitis is unlikely to be due to mumps. Case and surveillance studies have detected a host of other viruses in mumps-negative viral parotitis, but because of their weak association with viral parotitis, it has been difficult to establish causality. This case report is unique because a familial pair presented in tandem with different manifestations of an infection with the parainfluenza virus. These circumstances allowed the strong association of the parainfluenza virus with the mother’s croup to be transferred to the normally weak association of the parainfluenza virus with the son’s viral parotitis. This strongly inferred that the parainfluenza virus caused the patient’s viral parotitis and provides the best evidence to date of a virus other than mumps causing viral parotitis.
